# Comparison of serum ınflammatory parameters between spontaneous abortion and threatened abortion

**DOI:** 10.1590/1806-9282.20251941

**Published:** 2026-06-29

**Authors:** Mehmet Emre Peker, Duygu Uçar Kartal, Ufuk Atlıhan, Haydar Kaya, Can Ata

**Affiliations:** 1 Manisa Merkezefendi State Hospital – Manisa, Turkey.; 2 İzmir Buca Seyfi Demirsoy Training and Research Hospital – İzmir, Turkey.

**Keywords:** C-reactive protein, Abortion, spontaneous, Inflammation, Biomarkers, Blood cell count

## Abstract

**OBJECTIVE:**

Early pregnancy loss is a frequent reproductive health problem. Systemic inflammatory markers derived from complete blood count—neutrophil-to-lymphocyte ratio, platelet-to-lymphocyte ratio, and systemic inflammatory index—may reflect the inflammatory status present at clinical presentation. The aim of this study was to compare these indices between spontaneous abortion and threatened abortion.

**METHODS:**

This retrospective cross-sectional study included 146 women evaluated between January 2022 and April 2025. Patients were classified as spontaneous abortion (n=67) or threatened abortion (n=79) based on clinical and ultrasonographic findings. neutrophil-to-lymphocyte ratio, platelet-to-lymphocyte ratio, and systemic inflammatory index were calculated from complete blood counts. Group comparisons were performed using the Student's t-test or the Mann-Whitney U test. Blood samples were obtained at presentation, when the diagnosis of spontaneous abortion or threatened abortion had already been established; therefore, markers reflected concurrent inflammatory status rather than pre-diagnostic levels.

**RESULTS:**

The groups were similar regarding age, body mass index, gravida, parity, and gestational age (all p>0.05). Compared with threatened abortion, spontaneous abortion patients had significantly higher white blood cell and neutrophil counts, neutrophil-to-lymphocyte ratio, C-reactive protein, and systemic inflammatory index (all p<0.05). Although platelet-to-lymphocyte ratio values were numerically higher in the spontaneous abortion group, the difference was borderline and not statistically significant (p=0.058).

**CONCLUSION:**

Spontaneous abortion is associated with a stronger systemic inflammatory profile at presentation. Elevated systemic inflammatory index, neutrophil-to-lymphocyte ratio, and C-reactive protein levels reflect concurrent inflammation rather than definitive predictive or causal relationships.

## INTRODUCTION

Early pregnancy loss is defined as the termination of pregnancy before 20 weeks of gestation and occurs in approximately 10% of clinically recognized pregnancies^1^. About 15–25% of all pregnancies end in early loss, which is considered an important public health issue for women's health^2^. A large proportion of losses occurring before 12 weeks are associated with fetal chromosomal anomalies, and this risk increases significantly with advanced maternal age^3^. However, early pregnancy losses cannot be explained solely by chromosomal causes; immunologic, endocrine, infectious, thrombophilic, anatomic, and environmental factors also play important roles^4^. Despite a comprehensive evaluation of these factors, a definitive cause cannot be identified in approximately 50% of spontaneous abortion (SA) cases^5^.

Threatened abortion is a clinical condition characterized by vaginal bleeding and cramp-like pain in the first half of pregnancy, while the cervix remains closed and the pregnancy has the potential to continue^6^. Although many of these pregnancies progress normally, some evolve into SA. Therefore, threatened abortion (TA) is considered a critical turning point for both maternal and fetal outcomes. The role of the inflammatory response in the pathophysiology of this condition has been gaining increasing attention^7^.

In a normal pregnancy, despite the semi-allogeneic nature of the embryo, the maternal immune system must develop a balanced inflammatory response to maintain immune tolerance. A transient increase in proinflammatory cytokines (such as interleukin-1 beta [IL-1β], tumor necrosis factor-alpha [TNF-α], and interleukin-6 [IL-6]) during implantation and placentation is physiologically necessary. However, uncontrolled or excessive activation of these processes may impair trophoblast invasion and adversely affect placental development and uteroplacental circulation^8^. Consequently, this immunologic imbalance may predispose to early pregnancy loss.

The immunologic process of pregnancy can be reflected not only by cytokine levels but also by changes in the distribution of peripheral blood cells. During inflammation, neutrophils and platelets increase, whereas lymphocyte counts tend to decrease. This shift becomes evident in hematologic indices such as the neutrophil-to-lymphocyte ratio (NLR) and platelet-to-lymphocyte ratio (PLR)^9^. These ratios are considered indirect indicators of systemic inflammatory burden and have been reported to possess prognostic value in many obstetric and gynecologic conditions^10^. For example, significant elevations in NLR and PLR have been demonstrated in pathologies such as preeclampsia, preterm birth, gestational diabetes, and intrauterine growth restriction^11^.

Similarly, investigating inflammatory processes in early pregnancy loss through systemic markers has attracted interest in recent years. Some studies have reported that NLR and PLR are significantly higher in SA cases compared with controls, while these parameters change more modestly in TA^12,13^. These findings suggest that the magnitude and characteristics of the inflammatory response may differ between SA and TA.

Understanding the inflammatory mechanisms involved in the etiopathogenesis of SA is important for improving our understanding of the biological processes accompanying pregnancy loss. These hematologic indicators, which can be easily obtained from a complete blood count (CBC), represent low-cost and highly applicable biomarkers that may help describe the systemic inflammatory profile observed during early pregnancy loss. Nevertheless, studies directly comparing these parameters in TA and SA are limited.

Therefore, our study aimed to compare serum inflammatory parameters (such as NLR and PLR) in cases of SA and TA. In this way, we sought to explore the association between systemic inflammatory markers and early pregnancy loss at the time of clinical presentation. This approach may provide clinicians with practical, easily applicable biochemical tools for early diagnosis and risk stratification of pregnancy losses. In this manuscript, the term "spontaneous abortion" refers to early pregnancy loss with loss of embryonic or fetal viability, whereas "threatened abortion" refers to vaginal bleeding with preserved fetal cardiac activity. In this manuscript, the terms "early pregnancy loss" and "spontaneous abortion" are used interchangeably to describe pregnancy loss occurring before 20 weeks of gestation. The abbreviation SA refers to SA, while TA refers to TA characterized by vaginal bleeding with preserved fetal cardiac activity.

## METHODS

This retrospective cross-sectional study was conducted by reviewing the medical records of women who presented to the Department of Obstetrics and Gynecology between January 2022 and April 2025 and were evaluated during early pregnancy. Medical records of women who presented to the Department of Obstetrics and Gynecology between January 2022 and April 2025 and were evaluated during early pregnancy were reviewed. A total of 146 patients were included. Based on clinical and ultrasonographic findings, cases were categorized into two groups: the SA group (n=67), comprising cases with loss of embryonic or fetal viability before 20 weeks of gestation and with cervical dilatation; and the TA group (n=79), comprising cases with vaginal bleeding, a closed cervix, and fetal cardiac activity on ultrasound.

All patients were ≥18 years of age, had singleton intrauterine pregnancies, and had complete medical records. Exclusion criteria were as follows: presence of chronic inflammatory, autoimmune, or endocrine disease; acute infection; history of malignancy or hematologic disorder; use of corticosteroids or immunosuppressive/anti-inflammatory medications; multiple or ectopic pregnancy; and incomplete laboratory data. These criteria were intended to minimize potential confounders that might affect inflammatory parameters.

Demographic and obstetric characteristics—age, body mass index (BMI), gravida, parity, and gestational age—were obtained from the hospital information system. Laboratory parameters were derived from venous blood samples collected at presentation. White blood cell (WBC) count, neutrophil count, lymphocyte count, platelet count, and C-reactive protein (CRP) levels were analyzed in the same laboratory. Using these data, the NLR, PLR, and systemic inflammatory index (SII) were calculated. NLR was defined as neutrophils/lymphocytes, PLR as platelets/lymphocytes, and SII as (neutrophils×platelets)/lymphocytes. All blood samples were obtained at the time of hospital admission, when the diagnosis of SA or TA was established based on clinical and ultrasonographic findings. Therefore, inflammatory markers reflect the inflammatory status at presentation rather than pre-diagnostic levels.

### Statistical analysis

All analyses were performed using IBM SPSS Statistics for Windows, Version 27.0 (IBM Corp., Armonk, NY, USA). The distribution of continuous variables was assessed using the Kolmogorov-Smirnov test; normally distributed variables were expressed as mean±standard deviation (SD). For comparisons between the two groups, the Student's t-test was used for normally distributed variables and the Mann-Whitney U test for non-normally distributed variables. Categorical variables were analyzed using the chi-square test. A p-value of <0.05 was considered statistically significant for all analyses. No multivariable regression analysis was performed due to the exploratory design of the study and the limited sample size.

## RESULTS

**Figure 1:**
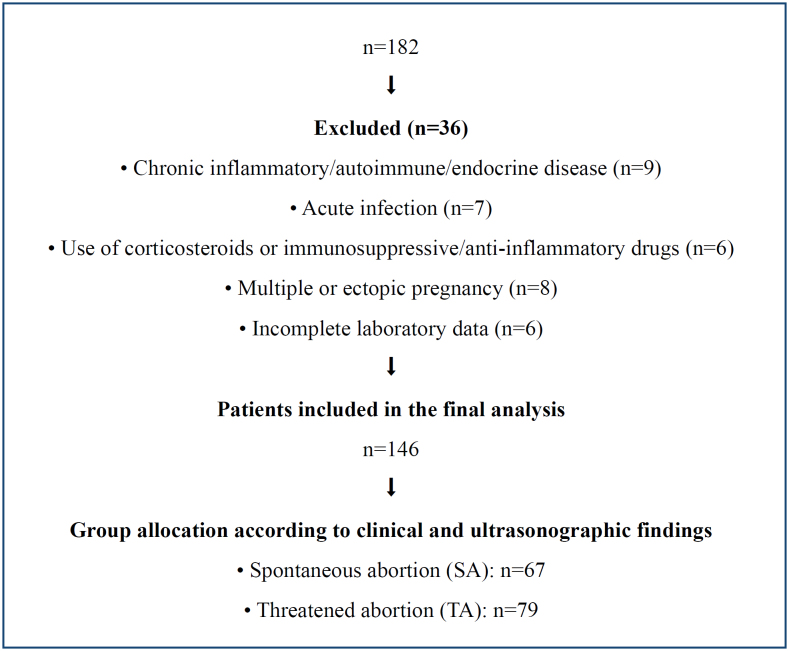
Flow diagram of patient selection and group allocation (January 2022–April 2025).

The flowchart of patient selection and group allocation is shown in Figure 1.

A total of 146 patients were included in the study: 67 in the SA group and 79 in the TA group. There were no statistically significant differences between the groups in terms of age, BMI, gravida, parity, or gestational age (all p>0.05). The mean age was 30.1±6.2 years in the SA group and 29.4±4.7 years in the TA group (p=0.45). Mean BMI values were 26.4±3.6 and 25.8±4.2 kg/m^2^, respectively (p=0.35). Gravida (2.2±1.4 vs. 2.3±1.3; p=0.65), parity (1.2±0.9 vs. 1.1±0.8; p=0.49), and gestational age (8.5±1.6 vs. 8.7±1.8 weeks; p=0.47) were also similar between the groups. These findings indicate that the study groups were homogeneous with respect to demographic and obstetric characteristics (Table 1).

**Table 1:** Comparison of demographic and obstetric characteristics between groups.

Parameters	Spontaneous abortion (n=67)	Threatened abortion (n=79)	p-value
Age (years)	30.1±6.2	29.4±4.7	0.45
Body mass index (kg/m^2^)	26.4±3.6	25.8±4.2	0.35
Gravida	2.2±1.4	2.3±1.3	0.65
Parity	1.2±0.9	1.1±0.8	0.49
Gestational age (weeks)	8.5±1.6	8.7±1.8	0.47

Note: Data are presented as mean±standard deviation (SD). Student's t-test was used for between-group comparisons.

When hematologic parameters were examined, WBC, neutrophil count, NLR, CRP, and SII values were significantly higher in the SA group than in the TA group. The mean WBC was 10.35±2.23×10^9^/L in the SA group versus 9.53±1.92×10^9^/L in the TA group (p=0.02). Neutrophil count was 6.72±1.98×10^9^/L versus 5.89±1.52×10^9^/L, respectively (p=0.006). Lymphocyte counts were similar between groups and did not differ significantly (1.99±0.67 vs. 2.15±0.67×10^9^/L; p=0.153).

Regarding inflammatory ratios, the NLR was significantly higher in the SA group (3.38±1.51) than in the TA group (2.74±1.11) (p=0.005). The PLR showed a trend but did not reach statistical significance (135.7±52.9 vs. 120.2±43.4; p=0.058). The C-reactive protein (CRP) level was significantly higher in the spontaneous abortion group than in the threatened abortion group (12.2±6.4 vs. 9.5±4.8 mg/L; p=0.005). The systemic inflammatory index (SII) was also markedly higher in the spontaneous abortion group (912±446 vs. 708±314; p=0.002) (Table 2).

**Table 2:** Comparison of hematologic and inflammatory parameters between groups.

Parameters	Spontaneous abortion (n=67)	Threatened abortion (n=79)	p-value
WBC (×10^9^/L)	10.35±2.23	9.53±1.92	0.02
Neutrophil count (×10^9^/L)	6.72±1.98	5.89±1.52	0.006
Lymphocyte count (×10^9^/L)	1.99±0.67	2.15±0.67	0.153
NLR	3.38±1.51	2.74±1.11	0.005
Platelet count (×10^9^/L)	270.1±53.1	258.5±47.2	0.169
PLR	135.7±52.9	120.2±43.4	0.058
CRP (mg/L)	12.2±6.4	9.5±4.8	0.005
SII (systemic inflammatory index)	912±446	708±314	0.002

Note: Data are presented as mean±standard deviation (SD). A p<0.05 was considered statistically significant. WBC: white blood cell; NLR: neutrophil-to-lymphocyte ratio; PLR: platelet-to-lymphocyte ratio; CRP: C-reactive protein; SII: systemic inflammatory index.

These results indicate that systemic inflammatory response is more pronounced in SA than in TA. The significantly higher levels of inflammatory markers—particularly NLR, CRP, and SII—suggest that inflammation may play an important role in the pathophysiology of spontaneous pregnancy loss.

Receiver operating characteristic (ROC) analysis demonstrated that SII showed the strongest statistical association with SA status at presentation (area under the curve [AUC]=0.764, 95%CI 0.685–0.843, p=0.002). An exploratory cut-off value of >812.5 was identified within this dataset, yielding a sensitivity of 72% and a specificity of 70%, indicating moderate diagnostic accuracy.

The NLR also showed significant discriminatory performance (AUC=0.718, 95%CI 0.632–0.804, p=0.005). At a cut-off value of >3.02, NLR discriminated SA with a sensitivity of 68% and specificity of 67%.

Similarly, CRP demonstrated moderate discriminative capacity (AUC=0.705, 95%CI 0.618–0.792, p=0.005). A threshold of >10.8 mg/L yielded a sensitivity of 65% and a specificity of 66%.

In contrast, WBC showed only limited discriminatory value (AUC=0.642, p=0.020), while the PLR did not demonstrate statistically significant discriminative ability (AUC=0.595, p=0.058), suggesting that PLR may not be a reliable marker for distinguishing SA from TA in this cohort (Table 3).

**Table 3:** Receiver operating characteristic analysis of inflammatory parameters for discriminating spontaneous abortion from threatened abortion.

Parameter	AUC	95%CI	Optimal cut-off	Sensitivity (%)	Specificity (%)
SII (systemic inflammatory index)	0.764	0.685–0.843	>812.5	72	70
NLR (neutrophil-to-lymphocyte ratio)	0.718	0.632–0.804	>3.02	68	67
CRP (C-reactive Protein, mg/L)	0.705	0.618–0.792	>10.8	65	66
WBC (white blood cell count, ×10^9^/L)	0.642	0.554–0.730	>9.95	62	60
PLR (platelet-to-lymphocyte ratio)	0.595	0.502–0.688	>128.4	58	55

AUC: area under the curve; SII: systemic inflammatory index; NLR: neutrophil-to-lymphocyte ratio; PLR: platelet-to-lymphocyte ratio; CRP: C-reactive protein; WBC: white blood cell.

## DISCUSSION

In this retrospective cross-sectional study, inflammatory parameters (e.g., NLR, PLR, SII) were compared between SA and TA cases, and WBC, neutrophil count, NLR, CRP, and SII values were found to be significantly higher in the SA group. These findings support an increase in systemic inflammatory response in early pregnancy losses and suggest that inflammation may play an important role in the underlying pathophysiologic process.

Recent research has shown that systemic inflammatory markers are closely associated with early pregnancy losses. In a study by Çallıoğlu et al., the SII and systemic inflammation response index were reported to be significant predictors of early pregnancy loss^14^. Similarly, Agaoglu et al. demonstrated that these parameters were markedly increased in recurrent pregnancy loss^15^. In our study, the significantly higher SII in the SA group parallels these results.

Studies focusing on classic inflammatory indicators—NLR and PLR—have yielded similar outcomes. In a systematic review, Hantoushzadeh et al. reported that NLR and PLR were significantly elevated in early pregnancy loss^16^. Yang et al. reported significantly higher WBC, neutrophil, and CRP levels among women experiencing early pregnancy loss^17^. Although PLR demonstrated a borderline increase in SA cases, this difference did not reach statistical significance; therefore, the role of PLR in distinguishing spontaneous from TA remains inconclusive in this cohort.

Likewise, Huang et al. emphasized that inflammatory indices derived from CBC (NLR, PLR, and MLR) may be valuable in predicting early pregnancy loss^18^. Erin et al. reported that SII is associated with SA and that higher SII levels may be linked to an increased likelihood of pregnancy loss^19^. In our study, SII values were also higher in the SA group, which is consistent with findings reported in the literature.

In a meta-analysis by Wang et al., PLR was proposed as an adjunct biomarker for assessing miscarriage risk^20^. However, in the study by Tekin et al., there were no significant differences in NLR, PLR, or SII between healthy pregnancies and those ending in abortion within the same women^21^. These discrepant findings may be explained by methodological differences such as sample characteristics, gestational age, stage of inflammatory response, and timing of blood sampling.

In a prospective study, Xiu et al. reported that serum inflammatory markers were strongly associated with adverse pregnancy outcomes^22^. Moreover, recent meta-analyses have increasingly clarified the role of NLR and SII in the pathophysiology of pregnancy loss; notably, inflammatory activation may impair trophoblast invasion and placental function^23,24^.

Taken together, inflammatory processes appear to play a prominent role in the etiopathogenesis of early pregnancy loss. The demographic similarity between our groups (age, BMI, gravida, parity, gestational age) supports the interpretation that differences in inflammatory parameters reflect true pathophysiologic differences. Because inflammatory markers were measured after the diagnosis of SA or TA had already been established, the observed elevations likely represent a consequence rather than a cause of pregnancy loss. Nonetheless, our study has limitations, including its retrospective design and the ­inability to fully control for subclinical infections and immunologic factors. Second, the study lacked multivariable adjustment for potential confounders such as subclinical infection, smoking status, history of prior miscarriage, or progesterone treatment, all of which may influence systemic inflammatory parameters. Therefore, residual confounding cannot be excluded. Finally, the single-center design and the relatively limited sample size may restrict the generalizability of the findings.

In conclusion, significantly higher SII, NLR, and CRP levels in SA underscore the importance of inflammation in the process of pregnancy loss. These findings indicate that SA is associated with a more pronounced systemic inflammatory profile at presentation. Prospective studies are required to determine whether these markers have any true predictive value. However, large-sample, prospective, multicenter studies are needed before these parameters can be fully integrated into clinical decision-making.

## Data Availability

The datasets generated and/or analyzed during the current study are available from the corresponding author upon reasonable request.
